# Investigation of a Quantitative Method for the Analysis of Chiral Monoterpenes in White Wine by HS-SPME-MDGC-MS of Different Wine Matrices

**DOI:** 10.3390/molecules20047359

**Published:** 2015-04-22

**Authors:** Mei Song, Ying Xia, Elizabeth Tomasino

**Affiliations:** Department of Food Science & Technology, Oregon State University, Wiegand Hall, Corvallis, OR 97331, USA; E-Mails: Mei.Song@oregonstate.edu (M.S.); xiay@onid.oregonstate.edu (Y.X.)

**Keywords:** monoterpenes, chiral, MDGC-MS, Riesling, Pinot Gris

## Abstract

A valid quantitative method for the analysis of chiral monoterpenes in white wine using head-space solid phase micro-extraction-MDGC-MS (HS-SPME-MDGC-MS) with stable isotope dilution analysis was established. Fifteen compounds: (S)-(−)-limonene, (R)-(+)-limonene, (+)-(2R,4S)-*cis*-rose oxide, (−)-(2S,4R)-*cis*-rose oxide, (−)-(2R,4R)-*trans*-rose oxide, (+)-(2S,4S)-*cis*-rose oxide, furanoid (+)-*trans*-linalool oxide, furanoid (−)-*cis*-linalool oxide, furanoid (−)-*trans*-linalool oxide, furanoid (+)-*cis*-linalool oxide, (−)-linalool, (+)-linalool, (−)-α-terpineol, (+)-α-terpineol and (R)-(+)-β-citronellol were quantified. Two calibration curves were plotted for different wine bases, with varying residual sugar content, and three calibration curves for each wine base were investigated during a single fiber’s lifetime. This was needed as both sugar content and fiber life impacted the quantification of the chiral terpenes. The chiral monoterpene content of six Pinot Gris wines and six Riesling wines was then analyzed using the verified method. ANOVA with Tukey multiple comparisons showed significant differences for each of the detected chiral compounds in all 12 wines. PCA score plots showed a clear separation between the Riesling and Pinot Gris wines. Riesling wines had greater number of chiral terpenes in comparison to Pinot Gris wines. Beyond total terpene content it is possible that the differences in chiral terpene content may be driving the aromatic differences in white wines.

## 1. Introduction

Monoterpene compounds are known to be important aroma compounds in aromatic white wines [1,2,3]. While it is noted that monoterpenes are important to aromatic white wine varieties, enantiomers of monoterpene compounds have been little explored in wine. Enantiomers are chiral molecules that are non-superimposable mirror images of each other. Many molecules are enantiomers, with one enantiomer being “active” and the other “inactive” such as with many pharmaceuticals. In wine, enantiomers of volatile aroma compounds have been found to have different perception thresholds and aroma descriptions [4]. For instance, (R)-(+)-limonene has a perception threshold of 200 ppb and an aroma of fresh, citrus and orange-like, while (S)-(−)-limonene has a perception threshold of 500 ppb and aroma described as harsh, turpentine-like, lemon note [5]. 

In nature many chiral compounds are enantio-pure or only found in one form. Unlike many other wine aroma compounds, terpenes are primarily derived from grapes, a natural source. These terpenes are present in bound forms in the grape (known as glycosides, as they are bound to sugars) and are not aromatically active. Once the terpenes are unbound, or free, they contribute to aroma [6]. There are some free forms in grapes but much of the free terpene content of wine is released during fermentation [6]. The free forms are released from their sugars due to enzymes, typically found in the yeast. However there are enzyme treatments (using glycosidase enzymes) that can increase the free terpene content of wine. Therefore the chiral terpene content of wine may be due to either viticulture or winemaking processes. It is entirely possible that these chiral compounds may be enantio-pure in wine or be present in all or some of their enantiomers. Due to their various properties and content in wine, the concentration of enantiomers or enantiomer excess has potential to explain relationships between wine chemistry, wine sensory, place of origin or different viticultural or winemaking processes that have been problematic or less explored in the past.

While the properties of chiral compound in wine are known, and mentioned above, measurement of the different enantiomers is challenging. The first record of enantiomeric separation in wine dates back to the mid 1800s with Pasteur’s separation of tartaric acid enantiomers [7,8,9]. Since this achievement chemists have looked for improved methods for the analysis and separation of chiral compounds. Derivatization of analytes with chiral reagents to form diastereomers and then chromatographically separated on achiral phases has been widely employed [10]. However this method has not been found to be successful for a range of chiral compounds, specifically chiral volatile aroma compounds.

Direct separation of enantiomeric compounds on chiral amide-based GC stationary phase (e.g., Chirasil-val) was demonstrated in the mid 1960s [11,12]. These columns allowed for excellent separation of amino acids but had limited application for analysis of most volatile compounds. With the introduction of chiral cyclodextrin-based GC stationary phases in the late 1970s and 1980s, chemists were provided with the ability to directly separate a large number of underivatized chiral compounds [13]. For example, several early studies demonstrated separations of cyclic and acyclic enantiomers with a range of functional groups including lactones, terpene hydrocarbons, carbonyls, alcohols, spiroketals, and oxiranes [14,15,16].

For the analysis of grapes and wines, cyclodextrin stationary phases have been used to establish the enantiomeric distribution of isomeric 3,4-dihydro-3-oxoedulans in Riesling wine [17], and solerone (5-oxo-4-hexanolide) and Riesling acetal (2,2,6,8-tetramethyl-7,11-dioxatricyclo[6.2.1.01,6]undec-4-ene) in brandy and Riesling [18]. Guth [19] separated the eight possible isomers of wine lactone (3a,4,5,7a-tetrahydro-3,6-dimethylbenzofuran-2(3H)-one) on a cyclodextrin stationary phase and determined that the predominate isomer occurring in Gewurztraminer wine was the 3S,3aS,7aR-isomer which has an intense sweet coconut-like aroma and an aroma threshold of 0.02 pg/L in air. The proposed study in this paper employs two different cyclodextrin columns in sequence using heart-cutting MDGC-MS to measure chiral terpenes in white wines, producing a more sensitive method for measurement of these compounds. Specifically compound stability over the course of analysis was investigated as was impact of wine matrix. Monoterpenes are important to white wine aroma, but not much is known about the contribution of chiral monoterpenes. Measurement of these chiral compounds has become more accessible as now measurement can be achieved with minimum sample preparation. The aim of this study was to produce a robust, reproducible and sensitive GC-MS based method to easily measure chiral monoterpenes. This method would then be used to investigate chiral monoterpene differences in Pinot Gris and Riesling wines. 

## 2. Results and Discussion

### 2.1. Separation of Chiral Mono-Terpenes in MDGC-MS

**Figure 1:**
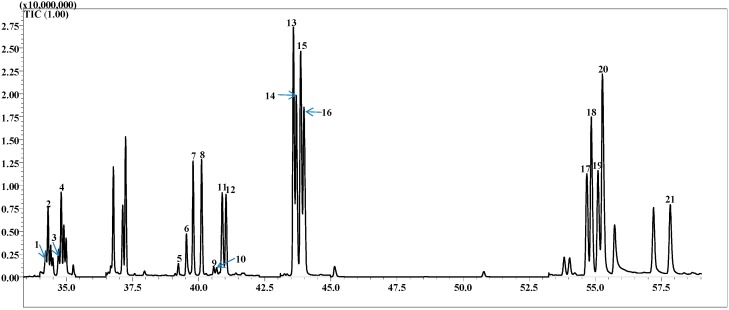
Separation of chiral monoterpenes and deuterium isotopes mixture using MDGC-MS. Numbers referred to the compounds: (1) d_3_-(S)-(−)-limonene, (2) (S)-(−)-limonene, (3) d_3_-(R)-(+)-limonene, (4) (R)-(+)-limonene, (5) (−)-(2S,4R)-*cis*-rose oxide, (6) (+)-(2R,4S)-*cis*-rose oxide, (7) (2R,5R)-(+)-*trans*-linalool oxide, (8) (2R,5S)-(−)-*cis*-linalool oxide, (9) (−)-(2R,4R)-*trans*-rose oxide, (10) (+)-(2S,4S)-*trans*-rose oxide, (11) (2S,5S)-(−)-*trans-*linalool oxide, (12) (2S,5R)-(+)-*cis*-linalool oxide, (13) d_3_-(R)-(−)-linalool, (14) (R)-(−)-linalool, (15) d_3_-(S)-(+)-linalool, (16) (S)-(+)-linalool, (17) d_3_-(−)-α-terpineol, (18) (−)-α-terpineol, (19) d_3_-(+)-α-terpineol, (20) (+)-α-terpineol, (21) (R)-(+)-β-citronellol.

A chromatogram of all 15 chiral monoterpenes and isotopes is found in Figure 1. All of the compounds showed good resolution. The elution order for linalool oxide and rose oxide isomers (standards were only available as isomer mixtures) were confirmed from previous methods investigating chiral compounds using the same column configuration as this method [20]. 

### 2.2. Validation of the Quantitative Method

#### 2.2.1. Linearity of Calibration Curve in Different Wine Matrix

Many reports have investigated the effect of non-volatile compounds on the perception of aroma compounds in wine, such as residual sugar, ethanol content, polyphenol, total acidity and so on [21,22,23]. Calibration curves performed in a synthetic matrix may not be representative of measurements taken in wines with diverse non-volatile compounds. In this study, two kinds of white wines with distinct different residual sugar contents were chosen as base wines for the calibration curves; Low wine (L) base for lower residual sugar content (3.7 g/L) and High wine (H) base for higher residual sugar content (64.1 g/L). These wines were found to appropriate to determine the effect of the non-volatile matrix on all wines measured in this study (data not shown). As can be seen in Table 1, there were some differences between the two kinds of calibration curves, especially for compounds of limonene and rose oxide isomers, thus two calibration curves were used in this study. Wines with corresponding residual sugar measurements were matched to the appropriate calibration curve. Wines with residual sugar content less than 19 g/L used the L calibration curve, while H calibration curve was used if the residual sugar content was greater than 19 g/L. The choice of 19 g/L as the dividing point was set based on spiked recovery study of wines with diverse residual sugar content (Table 2).

**Table 1:** Calibration curve information of 15 chiral monoterpenes in different de-aromatized wine matrices.

Compounds ^1^	ISTD ^2^	Ions Chosen ^3^ (*m/z*)	De-aromatized Low (L) Base ^5^			De-aromatized High (H) Base ^5^		
			L first ^4^	L middle ^4^	L last ^4^	H first ^4^	H middle ^4^	H last ^4^
			Slope	Slope	Slope	Slope	Slope	Slope
2	1	**107**, 121, 136	0.21 ^a^	0.12 ^b^	0.10 ^b^	0.14 ^a^	0.11 ^b^	0.10 ^b^
4	2	**107**, 121, 136	0.23 ^a^	0.12 ^b^	0.10 ^b^	0.12 ^a^	0.12 ^a^	0.11 ^a^
5	3	**139**, 69, 83	0.14 ^a^	0.33 ^c^	0.25 ^b^	0.21 ^a^	0.32 ^c^	0.29 ^b^
6	3	**139**, 69, 83	0.13 ^a^	0.28 ^c^	0.24 ^b^	0.19 ^a^	0.30 ^b^	0.28 ^b^
7	3	**94**, 93, 111	0.01 ^a^	0.02 ^a^	0.02 ^a^	0.01 ^a^	0.02 ^a^	0.03 ^a^
8	3	**94**, 93, 111	0.01 ^a^	0.02 ^a^	0.02 ^a^	0.01 ^a^	0.02 ^a^	0.03 ^a^
9	3	**139**, 69, 83	0.09 ^a^	0.17 ^b^	0.15 ^b^	0.11 ^a^	0.14 ^b^	0.16 ^b^
10	3	**139**, 69, 83	0.07 ^a^	0.13 ^b^	0.13 ^b^	0.09 ^a^	0.11 ^ab^	0.13 ^b^
11	3	**94**, 93, 111	0.01 ^a^	0.02 ^a^	0.02 ^a^	0.01 ^a^	0.02 ^a^	0.03 ^a^
12	3	**94**, 93, 111	0.01 ^a^	0.02 ^a^	0.02 ^a^	0.01 ^a^	0.02 ^a^	0.03 ^a^
14	3	**121**, 93, 136	0.01 ^a^	0.01 ^a^	0.01 ^a^	0.01 ^a^	0.01 ^a^	0.01 ^a^
16	4	**121**, 93, 136	0.01 ^a^	0.01 ^a^	0.01 ^a^	0.01 ^a^	0.01 ^a^	0.01 ^a^
18	5	**59**, 81, 121	0.07 ^a^	0.06 ^a^	0.06 ^a^	0.06 ^a^	0.06 ^a^	0.06 ^a^
20	6	**59**, 81, 121	0.05 ^a^	0.05 ^a^	0.05 ^a^	0.05 ^a^	0.05 ^a^	0.05 ^a^
21	6	**95**, 109, 123	0.03 ^a^	0.04 ^a^	0.03 ^a^	0.03 ^a^	0.03 ^a^	0.03 ^a^

^1^ The numbers for compounds are the same with Figure 1; ^2^ ISTD=internal standard; ^3^ Numbers in bold are quantification ions; ^4^ R^2^ for each curve is 0.99; ^5^ The one way ANOVA test was only performed among fiber age inside each matrix. Letter superscripts within each matrix are significantly different from one another at *p* < 0.05 by Tukey’s HSD test.

**Table 2:** LOD, LOQ, percent spiked recovery, reproducibility and standard stability for wine samples with different matrix.

Compounds	LOD (ug/L)	LOQ (ug/L)	Spiked Recovery (%)						Riesling Reproducibility (RSD) ^g^	Pinot Gris Reproducibility (RSD ) ^h^	Standards Stability (RSD) ^i^
			in PG Dry ^a^	in PG Medium Dry ^b^	in RS Dry ^c^	in RS Medium Dry ^d^	in RS Medium Sweet ^e^	in RS Sweet ^f^			
2	0.10	0.34	106	94	90	87	103	114	15.60	0.00	12.1
4	0.08	0.27	104	93	93	111	106	112	15.91	0.00	11.1
5	0.0002	0.001	101	129	119	135	137	119	0.00	0.00	15.2
6	0.0003	0.001	99	112	132	103	125	105	12.98	0.00	13.0
7	0.73	1.09	72	102	90	118	110	97	18.35	17.27	10.7
8	0.44	0.70	72	101	93	112	113	97	18.13	14.29	11.0
9	0.001	0.003	99	116	126	113	120	123	0.00	0.00	12.9
10	0.0006	0.002	94	102	114	118	110	114	15.57	0.00	12.8
11	0.28	0.93	76	103	103	116	118	100	15.12	0.00	15.6
12	0.33	1.11	72	100	97	115	114	96	0.00	15.26	12.8
14	0.03	0.12	101	98	106	101	102	103	0.00	0.00	9.5
16	0.08	0.25	102	100	109	98	101	102	12.57	0.00	9.5
18	0.19	0.62	100	96	112	104	107	102	13.35	14.21	4.3
20	0.15	0.49	98	95	106	101	103	102	14.85	16.00	4.8
21	0.02	0.08	98	103	96	81	108	107	15.60	11.01	15.0

^a^ Three “dry” style Pinot Gris wines; ^b^ Four “medium dry” style Pinot Gris wines; ^c^ One “dry” style Riesling wine; ^d^ Four “medium dry” style Riesling wine; ^e^ Seven “medium sweet” style Riesling wine; ^f^ Seven “sweet” style Riesling wine; ^g^ 2011 dry Riesling, 10.8% alcohol content (*v*/*v*), 0.58 g/L residual sugar; ^h^ 2013 Pinot Gris, 11.8% alcohol content (*v*/*v*), 16.43 g/L residual sugar; ^i^ The fourth level of Standards.

One of the differences observed between the L and H calibration curves was the slope. A lower slope for 2 and 4 (limonene isomers) and higher slope for 5, 6, 9 and 10 (rose oxide isomers) was found in the H base calibration curve compared with the L base calibration curve. Rose oxide isomers seemed more easily released from the diluted wine solution with higher residual sugar content, while limonene isomers were retained longer in the same wine base. Other work has shown that high concentrations of fructose in model wine solutions have strong odorant retaining effects in headspace extraction [24]. However, another study showed the extraction of esters was not affected by sugar content in a varying saccharose content (0–200 g/L) synthetic wine using HS-SPME-HRGC [25], suggesting that the impact of sugar concentration on extraction for HS-SPME measurements varies depending on the volatile compound being measured. Our results showed that the measurement for concentration of chiral monoterpenes in wine with a high residual sugar would not be accurate if a calibration curve from a dry wine were utilized to calculate the concentration of terpenes, especially for limonene and rose oxide isomers. This suggested a strong matrix effect on the investigated compounds.

Several other method factors were investigated to ensure accurate and repeatable measurements of the chosen chiral monoterpenes. HS-SPME was used as it is an inexpensive and reproducible method to measure volatile compounds [26,27,28]. In our study, single fiber repeatability was investigated in both wine bases. Calibration curves were divided into three parts, based on the age (or usage) of a new fiber; the beginning (first 8–10 days of fiber life), middle use (within 8–13 days of fiber life) and at the last life of fiber (last 10 days of fiber life). In total, six calibration curves were plotted in terms of wine matrix and fiber age over 300 injections; L first, L middle, L last, H first, H middle and H last. A significant change in slope for compounds 2, 4, 5, 6, 9 and 10 was noted from first to last intervals for both wine bases (Table 1). Interestingly, limonene isomers (2 and 4) had decreasing affinity for the SPME fiber in single fiber durability. Rose oxide isomers (5, 6, 9 and 10) showed a greater affinity for the fiber during the middle interval, in comparison with the decreasing affinity noted for other compounds. 7, 8, 11, 12, 14, 16, 18, 20 and 21were not affected by the life of the SPME fiber. The reason for the poor repeatability of single fiber was not clear. It was reported that SPME fibers suffer from some weakness, such as limited lifetime and sample carryover [29]. The carry over effect had been eliminated in our study by re-desorption in the heater port for 10 min, lengthening this time did not improve compound affinity over time. One report mentioned poor reproducibility from one fiber to another, and suggested a new calibratation each time the fiber was changed [30]. To eliminate the error due to single fiber variance, chemical composition was determined using calibration curves from the same time period.

#### 2.2.2. Limit of Detection (LOD), Limit of Quantitation (LOQ), Wine Reproducibility and Internal Standards Stability

LOD and LOQ data are reported in Table 2. The LOQ ranges from 1 ng/L to 1.11 μg/L and these limits are lower than the known olfactory thresholds for the measured compound found in Section 3.1, Table 5. The detected LOQ (µg/L) values are also lower than previous methods measuring chiral terpenes [2,31,32]. Wine reproducibility values varied around 15% (RSD %) for all compounds in Riesling and Pinot Gris wines. Reproducibility of 14, 16, 18 and 20 were the best of all compounds, with an RSD of less than 10%. This study showed that all the terpenes in the standards and wines were stable and did not degrade or react under the chosen storage conditions (−18 °C).

#### 2.2.3. Accuracy

The majority of the spiked recoveries fell within 80%–120%, although there were some exceptions (Table 2). Compounds 7, 8, 11 and 12 in PG dry style were about 70% and 5 and 9 in most wine styles was approximately 130%. As mentioned before in Table 1, compounds 7, 8, 11 and 12 had greater retention in L base, which may explain the low calculated recovery. Concentrations of 7, 8, 11 and 12 from Pinot Gris dry white wine were adjusted to 100% by a recovery factor (multiply by 1.4 for 7, 8 and 12; by 1.3 for 11). A possible explanation for the spiked recoveries for 5 and 9 (~130%) may be due to the very low concentrations measured. Reports show that systematic errors may be present for some monoterpenes at very low concentration, especially close to the LOQ if the internal standard was not analogous to that compound being quantified, thus leading to non-linear responses [33] and recovery values higher than 100% [34]. Compounds 5 and 9 (rose oxide isomers) were calculated using d_3_-linalool as the internal standard as a deuterium labeled rose oxide isotope was not available.

#### 2.2.4. Temperature Stability

The temperature stability of the chiral monoterpenes at key steps of the method was investigated as studies have shown that these compounds may degrade at high temperatures [35,36]. The extraction temperature during headspace sampling and the injector temperature were investigated for seven of the chiral monoterpenes (Table 3). 

**Table 3:** Average variation (peak area ratio) of seven monoterpene standards based on injection and extraction temperature.

Standards	Injector Temperature (°C)			Extraction Temperature (°C)	
	200	230	250	40	60
20	91.5	94.2	93.8	94.9	93.8
14	97.1	97.9	97.9	98.7	97.9
21	98.5	100.0	99.9	100.0	99.9
4	100.0	100.0	100.0	100.0	100.0
2	88.7	85.8	89.5	89.6	89.5
6	15.8	15.4	14.5	14.3	14.5
8	69.0	62.8	64.7	64.5	64.7

The stability of the terpenes at temperatures of 200 °C, 230 °C and 250 °C were determined. The range of injector temperatures was chosen because they are higher than the boiling point of all compounds and lower than the column maximum temperature. T-test and one way ANOVA were performed and no significant differences were found due to injector and extraction temperatures (α = 0.05, Table 3). The results showed that the chosen temperatures did not impact the adsorption of the compounds onto the SPME fiber or any degradation of the compounds when injected into the GC.

### 2.3. Wine Analysis 

All of the measured chiral monoterpenes were found in 12 white wines except for 5, 9 and 10. P refers to Pinot Gris wines and R refers to Riesling wines. The concentrations of all the compounds detected in investigated wines, except 14 and 16, were below perception threshold detected in air or ethanol solutions (Table 4). ANOVA with Tukey multiple comparisons showed significant differences for all of the detected chiral compounds. 7, 8, 12, 18 and 20 were detected in all 12 white wines. Compound 21 was not detectable in R2 wine. 14 and 16 were found in all six Riesling wines and three Pinot Gris wines. Interestingly, 5 and 11 were only detected in five and six wines respectively. The presence of only one rose oxide isomer (5) is consistent with another report of (−)-*cis*-rose oxide present at high enantiomeric excess in grape musts [4]. These results suggest that the profile of chiral mono-terpenes may have the potential to differentiate diverse wines. 

**Table 4:** Multiple Comparisons (Tukey) of the average concentration of 12 chiral monoterpenes for 12 white wines.

**(2) *S*-(−)-limonene (*p* < 0.0001)**			**(4) *R*-(+)-limonene (*p* < 0.0001)**			**(5) Rose Oxide Isomer (*p* < 0.0001)**		
**Wine**	**Conc (µg/L)**	Conf. Int. *	**Wine**	**Conc (µg/L)**	Conf. Int. *	**Wine**	**Conc (µg/L)**	Conf. Int. *
R6	11.88	A	R6	10.94	A	P6	0.33	A
R4	10.56	AB	R4	9.1	AB	R5	0.3	A
R3	9.19	AB	R3	8.22	AB	R4	0.11	AB
R5	8.61	AB	R5	7.24	AB	P5	0.02	AB
R1	4.29	ABC	R1	3.62	ABC	P1	0.02	AB
R2	2.79	ABCD	P5	1.71	ABCD	P2	nd	AB
P5	2.33	ABCD	P6	1.39	ABCD	P3	nd	AB
P6	2.19	ABCD	P1	1.29	ABCD	P4	nd	AB
P1	1.57	ABCD	P2	0.75	ABCD	R1	nd	AB
P2	0.83	ABCD	R2	0.21	ABCD	R2	nd	AB
P3	nd	ABCD	P4	nd	ABCD	R3	nd	AB
P4	nd	ABCD	P3	nd	ABCD	R6	nd	AB
**(7) Linalool Oxide Isomer (*p* < 0.001)**			**(8) Linalool Oxide Isomer (*p* < 0.001)**			**(11) Linalool Oxide Isomer (*p* = 0.000)**		
**Wine**	**Conc (µg/L)**	Conf. Int. *	**Wine**	**Conc (µg/L)**	Conf. Int. *	**Wine**	**Conc (µg/L)**	Conf. Int. *
R5	44.3	A	R5	34.28	A	R5	14.42	A
R2	42.69	AB	R2	22.25	AB	R4	6.7	AB
R4	34.5	ABC	R1	20.03	AB	P5	5.06	AB
R1	29.01	ABC	R4	18.28	AB	R1	4.2	AB
R3	25.42	ABC	R3	16.17	AB	P6	4.05	AB
P1	9.85	ABC	R6	13.62	AB	P4	3.4	AB
P4	9.73	ABC	P4	10.66	AB	P1	nd	AB
P3	9.69	ABC	P3	9.89	AB	P2	nd	AB
P6	8.9	ABC	P5	9.78	AB	P3	nd	AB
R6	8.37	ABC	P1	9.25	AB	R2	nd	AB
P5	4.99	ABC	P6	8.58	AB	R3	nd	AB
P2	4.69	ABC	P2	7.61	AB	R6	nd	AB
**(12) Linalool oxide isomer (*p* < 0.0001)**			**(18) (−)-α-Terpineol (18) (*p* < 0.0001)**			**(16) S-(+)-Linalool (*p* < 0.0001)**		
**Wine**	**Conc (µg/L)**	Conf. Int. *	**Wine**	**Conc (µg/L)**	Conf. Int. *	**Wine**	**Conc (µg/L)**	Conf. Int. *
R5	17.82	A	R6	58.84	A	R6	46.49	A
R4	14.86	AB	R3	30.61	AB	R3	37.99	AB
R2	14.23	AB	R4	26.93	AB	R4	29.17	ABC
R1	10.96	ABC	R2	12.77	AB	R2	13.47	ABC
R3	10.35	ABCD	P6	12.33	AB	P6	12.59	ABC
P6	5.1	ABCD	P5	12	AB	P5	12.51	ABC
P4	5.03	ABCD	R1	11.82	AB	R1	11.54	ABC
P5	4.35	ABCD	R5	7.27	AB	R5	9.67	ABC
P3	4.26	ABCD	P1	5.26	AB	P1	5.44	ABC
P2	3.69	ABCD	P4	nd	AB	P4	nd	ABC
P1	3.62	ABCD	P3	nd	AB	P2	nd	ABC
R6	1.02	ABCD	P2`	nd	AB	P3	nd	ABC
**(20) (+)-α-Terpineol (*p* < 0.0001)**			**(18) (−)-α-Terpineol (18) (*p* < 0.0001)**			**(21) R-(+)-β-citronellol (*p* < 0.0001)**		
**Wine**	**Conc (µg/L)**	Conf. Int. *	**Wine**	**Conc (µg/L)**	Conf. Int. *	**Wine**	**Conc (µg/L)**	Conf. Int. *
R4	65.52	A	R4	76.12	A	P2	8.3	A
R3	54.09	AB	R6	59.47	AB	P6	5.78	AB
R6	50.21	AB	R5	53.57	ABC	R3	5.72	AB
R5	47.6	AB	R3	51.66	ABCD	P5	5.38	ABC
R1	31.08	ABC	R1	35.71	ABCDE	P4	4.64	ABCD
R2	27.98	ABC	R2	30.28	ABCDE	R4	4.03	ABCDE
P1	15.12	ABC	P1	18.87	ABCDE	P1	3.85	AB DEF
P5	14.07	ABC	P5	17.72	ABCDE	R5	3.75	AB DEF
P6	13.57	ABC	P3	17.29	ABCDE	R1	3.62	ABC EF
P4	13.45	ABC	P4	16.77	ABCDE	R6	2.99	ABCD F
P2	5.79	ABC	P6	15.45	ABCDE	P3	2.97	ABCD F
P3	5.22	ABC	P2	7.7	ABCDE	R2	nd	ABCD G

* Numbers with different superscripts within each compound are significantly different from one another at *p* < 0.05 by Tukey’s HSD test.

**Figure 2:**
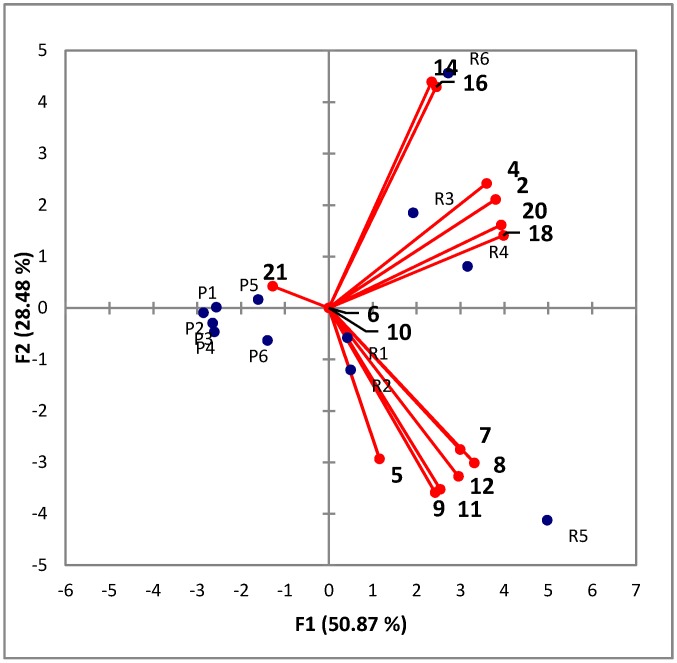
PCA plot of 12 white wines on concentration of chiral mono-terpenes.

PCA of the chiral monoterpenes showed a clear separation between the Riesling and Pinot Gris wines, with Riesling wines containing more chiral terpenes than Pinot Gris wines (Figure 2). While the Pinot Gris wines all grouped in the same relative area, the Riesling wines were further separated into two groups on F2. Specifically, half of the wines were correlated with 5, 6, 7, 8, 9, 10, 11, and 12 and the other half correlated to 2, 4, 14, 16, 18 and 20. 

It would be interesting to determine the origins of the differences in chiral monoterpenes. The ANOVA and PCA results suggest there is some difference in grape varieties as the Pinot Gris and Riesling wines group in a similar area. This differentiation by grape variety was expected, as it is known that the precursors to these terpenes are formed in the grape [6,37]. It was anticipated that the Pinot Gris wines would have lower concentrations and a less complex composition of monoterpenes than the Riesling wines, as Riesling wines are considered to be a more aromatic varietal. The further separation on the right side of the PCA plot, namely the vertical separation of Riesling, may be due to a number of factors. There are many mechanisms for release of terpenes from their bound forms including enzyme additions, pH of juice and wine, yeast strain used for fermentation, fermentation temperature and other viticulture and winemaking practices [38,39,40,41,42]. The further separation of the Riesling wines is most likely due to viticultural or winemaking practices. Specifically R1, R2 and R5 are characterized as higher concentrations of the four linalool oxide isomers and three of the rose oxide isomers. R3 and R4 are best characterized by limonene and α-terpineol isomers and R6 characterized by linalool isomers. 

The higher concentrations of linalool-oxides in the R1, R2 and R5 may correlate to ripeness in the grapes prior to fermentation, as these oxides are typically formed from oxidation and cyclization of linalool [43]. Alternatively linalool oxides are also produced from *Botrytis cinerea* metabolism, and this may be the defining factor between the Riesling wines [44], although none of the wines chosen were late harvest or styles of Riesling associated with *Botrytis cinerea*. However those wines with higher levels of linalool oxides isomers also contained high levels of rose oxides isomers. Formation of rose oxide isomers is from a different source, namely yeast metabolism. There are two different reductive pathways known in yeast that result in rose oxide formation [45]. The limonene and α-terpineol found in R3 and R4 is quite interesting as acidic environments, such as in wine, result in the formation of α-terpineol from limonene [46]. Therefore it was anticipated that wines with higher concentrations of α-terpineol would have lower concentrations of limonene isomers. But α-terpineol is also one of the most abundant terpenes produced by yeast [47], therefore the terpene content of these two Riesling wines may be due to a combination of factors. The high levels of linalool in R6 may also be due to several factors, such as a high level of linalool-glycosides in the grapes and then of release of this into the free form. But it could also be related to the low transformation of linalool after fermentation resulting in retention of more linalool than the other wines. As stated before linalool degrades to linalool oxides in the presence of oxygen, but it may also be degraded by *Botrytis cinerea* [48]. It has also been found that higher levels of caffeic and gallic acid can stop the degradation of linalool in wine [49]. Therefore the nonvolatile composition, which was not investigated beyond sugar content in this study, may be more favorable for linalool retention.

Finally it is of great interest to determine if the differences in the chiral monoterpene content of these wines is also present in sensory perception. We have stated earlier in the paper that all of these compounds are present at levels below their known perception threshold, but current perception thresholds may not be realistic in wine, as the solution they are tested in plays an important role and most perception thresholds are measured in water or a water and ethanol solution [50,51,52]. To add to this complexity it is known that the perception threshold of some monoterpenes, specifically linalool, changes when it is in a mixture with other monoterpenes [53]. Therefore further research investigating the sensory impact of these compounds is important. It will be able to determine how the compound impacts aroma individually and then in combination with other chiral terpenes. 

## 3. Experimental Section 

### 3.1. Chemicals

**Figure 3:**
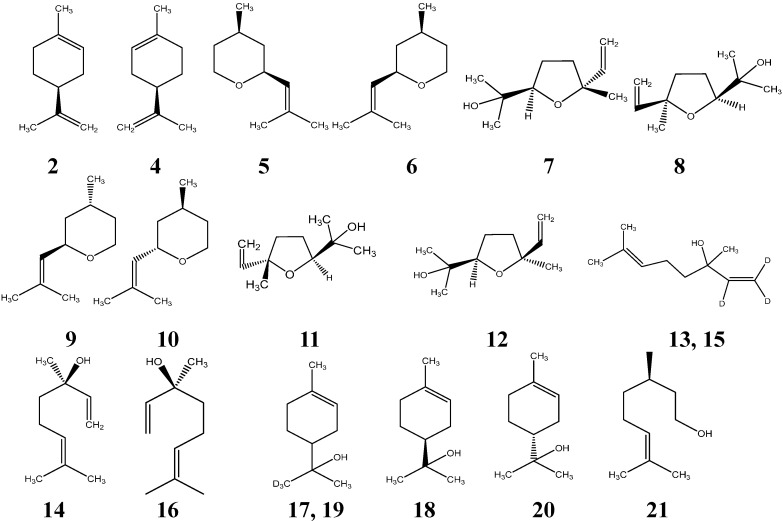
Chemical structures of the chiral monoterpene isomers.

The following standards were obtained from Sigma Chemical Co. (St. Louis, MO, USA): (*S*)-(−)-limonene, (*R*)-(+)-limonene, (−)-rose oxide, linalool oxide, linalool, α-terpineol, and (*R*)-(+)-β-citronellol (Table 5). The chemical structure of the standards were showed in Figure 3. D_3_-(±)-α-terpineol and d_3_-(±)-linalool were purchased from CDN Isotopes (Pointe-Claire, QC, Canada). D_3_-(±)-limonene was not available from a commercial source. Synthesis information can be found in Section 3.2. Other chemicals, including methylvinyl ketone (99%, CAS# 78-94-4), isoprene (CAS# 78-79-5), aluminum chloride (CAS# 7446-70-0), magnesium sulfate (CAS# 7487-88-9), d_3_-methyl-triphenylphosphonium iodide (95 atom %D, CAS# 1560-56-1), and sodium amide (98%, CAS# 7782-92-5) were purchased from Sigma Chemical Co. Sodium sulfate (anhydrous, CAS# 7757-82-6) was from Mallinckrodt AR^®^ (St. Louis, MO, USA); Potassium carbonate (anhydrous, CAS# 584-08-7) from EMD (Billerica, MA, USA). Organic solvents used were HPLC grade, absolute ethyl alcohol (anhydrous) was from Pharmco-AAPER (Vancouver, WA, USA), dichloromethane and *n*-hexane (95%) were from EMD, ethyl ether (anhydrous) was from Macron Fine Chemicals (Center Valley, PA, USA). Milli-Q water was obtained from a Millipore Continental water system. Residual sugar of each wine was analyzed in duplicate according to the revised Rebelein method [54]. Alcohol content was analyzed using an Alcolyzer Wine M (Anton Paar GmbH, Graz, Austria).

### 3.2. D_3_-Limonene Synthesis

#### 3.2.1. Synthesis of 4-Acetyl-1-methylcyclohexane

Dichloromethane (3 mL) was placed in a 5 mL beaker on an ice bath. Methylvinyl ketone (500 µL) and isoprene (500 µL) were slowly pipetted into the beaker, and a small spatula tip of anhydrous AlCl_3_ was added. This solution was stirred for 2 h at room temperature. The resulting mixture was diluted with diethyl ether (5 mL) and then washed with 10% Na_2_SO_4_ solution (x2). The upper layer was separated and dried with magnesium sulphate. The resulting compound was checked with GC-MS. The yield of 4-acetyl-1-methylcyclohexane was around 1 mL.

**Table 5:** Odor descriptors, purity, CAS# and perception threshold (µg/L) for chemical standards.

Compounds	Odors	Purity (%)	CAS No. ^b^	Perception Threshold (µg/L)
1 ^a^		N/A	N/A	N/A
2	Harsh, turpentine-like, lemon note [4]	89.6	5989-54-8	500 [4]
3 ^a^		N/A	N/A	N/A
4	Fresh, slightly orange note [4]	99.0	5989-27-5	200 [4]
5	Herbal, green, floral, hay green, earthy, heavy [31]	99.0	16409-43-1	50 [31,32]
6	Floral, green, clean, sharp, light, rose green [31]	99.0	16409-43-1	0.5 [31,32]
7	Earthy, leafy [33]	97.0	60047-17-8	3000–4000 [34]
8	Stronger earthy, leafy [33]	97.0	60047-17-8	3000–4000 [34]
9	Floral green, green herbal, minty, fruity [31]	99.0	16409-43-1	160 [31,32]
10	Herbal, green, floral, fruity, herbal, rose, citrus (bitter peel) [31]	99.0	16409-43-1	80 [31,32]
11	Sweet, floral, creamy [33]	97.0	60047-17-8	3000–4000 [34]
12	Sweet, floral, creamy [33]	97.0	60047-17-8	3000–4000 [34]
13		99.4	1216673-02-7	N/A
14	Woody, lavender [31]	99.0	78-70-6	0.8 [35]
15		99.4	1216673-02-7	N/A
16	Sweet, petigrain [31]	99.0	78-70-6	7.4 [35]
17		99.9	203633-12-9	N/A
18	Coniferous odor, tarry, cold pipe like [36]	96.0	98-55-5	300,000 [37]
19		99.9	203633-12-9	N/A
20	Heavy floral lilac-like odor [36]	96.0	98-55-5	300,000 [37]
21	Slightly oily light rosy-leafy, petal-like odor with irritating top note [38]	98.0	1117-61-9	50 [38]

^a^ synthesized in the lab; ^b^ compounds with the same CAS No. were from the isomer mixture.

#### 3.2.2. D_3_-Limonene Synthesis

Anhydrous ethyl ether (10 mL) was added to a three-necked flask with continual nitrogen blowing. With vigorous stirring, 4-acetyl-1-methylcyclohexene (1 mL) and d_3_-methyltriphenylphosphonium iodide (around 0.5 g) was added to the ethyl ether. The flask was closed with its caps and stirred vigorously for 10 min, after that, around sodium amide solution (2.5–3 g) dissolved in anhydrous ethyl ether (10 mL) was slowly added. The mixture was stirred vigorously for 15 min. The solution was allowed to cool to room temperature and transferred to a separatory funnel. The mixture was diluted with hexane (30 mL) and washed with 10% aqueous solution K_2_CO_3_ (10 mL ×2). The upper layer was retained and dried with 10% Na_2_SO_4_ (10 mL ×2), then dried with Mg_2_SO_4._ The resulting compound was concentrated by gently boiling the organic layer on a hot plate. The final compound was thick residue with yellow color and orange aroma. Three ions were different (71, 124, and 139) in comparison to regular limonene (68, 121, and 136), showing that hydrogen was replaced by deuterium in three places.

### 3.3. Sample Preparation

All wine samples were diluted immediately prior to analysis. Each wine (0.9 mL) was added to milli-Q water (8.06 mL) in 20 mL amber glass, screw cap vials, 22.5 × 75.5 mm, followed by the composite isotopically-labelled internal standard solution (40 µL). The volumes used were equivalent to a 10-fold dilution of the wine sample [55]. Sodium chloride (4.5–5.0 g) was added to the SPME vial and vials were tightly capped. Samples were then incubated initially for 10 min at 60 °C, during which time the vial was agitated at 500 rpm (5 s on, 2 s off). The sample was extracted for 50 min with no further agitation. The fiber was then injected into the first GCMS for 10 min at 250 °C followed by further conditioned in an NDL heater for 10 min at 250 °C.

### 3.4. Solid Phase Micro-Extraction Coupled with MDGC-MS

Three-phase Stableflex SPME fibers (50/30 µm DVB/CAR/PDMS, 2 cm, 24 Ga) were purchased from Supelco (Poznań, Poland). The fiber was conditioned at 250 °C for 1 h before analysis. All samples were extracted at 4 °C using a Shimadzu AOC-5000 plus auto-sampler fitted with a stack cooler set. Heart cut-MDGC-MS analyses were performed using a Shimadzu GC-2000 plus with a split/splitless injector coupled to a Shimadzu QP 2010 GC-mass spectrometer using a Dean switch. The first GC column was a RtX-wax, 30 m in length, 0.25 mm ID, and 0.5 µm of film thickness (Crossbond^®^ Carbowax^®^ polyethylene glycol, Restek Corporation, Bellefonte, PA, USA). Method parameters for the first GC oven were as follows; injector temperature at 230 °C. The column oven was held at 65 °C for 3 min, and then increased to 145 °C at 4 °C·min^−1^, at which the temperature was kept constant for 10 min, then further increased to 230 °C at 4 °C·min^−1^ and held at this temperature for 15 min. Flow control mode was set using pressure mode at a constant 235 kpa, switching pressure was 200 kpa. The cut windows for further separation on second GC column were: 7.85–8.50 min; 12.00–14.00 min; 14.25–15.80 min; 15.85–17.25 min, 18.50–21.25 min; 22.00–26.00min; 26.75–30.00 min, 33.00–38.00 min, 38.50–45.00 min. The second GC contained two columns connected in sequence. An Rt®-βDEXsm connected with Rt^®^-βDEXse (alkylated β-cyclodextrin in cyanopropyl-dimethyl polysiloxane, Restek Corporation), 60 min length, 0.25 mm ID, and 0.25 µm of film thickness. The second oven program began at the same time as the first as is as follows; the column oven was held at 40 °C for 10 min, then increased to 125 °C at 3.0 °C·min^−1^ holding for 10 min, followed by an increase of 3.0 °C·min^−1^ to 135 °C, then further increased to 170 °C at 2.0 °C·min^−1^, held for 2 min, finally increased to 230 °C at 15.0 °C min^-1^ and held at this temperature for 8 min. The total run time was 83.17 min. GCMS transfer line temperature was 230 °C; ion source temperature was 200 °C. Spectra were acquired using electron impact ionization (EI, 70 eV) in a full scan mode from 3 min to 83 min with scan range of m/z 33–303 Da at 0.20 sec event time. Detector was run at a variable gain factor for each compound from 0.80 to 1.35.

Identification of all chiral monoterpenes was based on the comparison of retention time and mass spectra with authentic standards and NIST11 database. Quantitation of all compounds was based on calibration curves calculated from peak area ratios (peak area of the monoterpene standard/peak area of the corresponding isotope standard) were plotted against the concentration ratios (monoterpene standard/the corresponding isotope standard) for six series of monoterpene concentrations. 

### 3.5. Validation of the Quantitative Method

#### 3.5.1. Linearity of Calibration Curve in Different Wine Matrix

All 15 chiral monoterpenes were quantified using six point calibration curves. Two de-aromatized wines were prepared as the base wine: one was a 2012 Pinot Grigio, alcohol content 12.3%, residual sugar 3.7 g/L, defined as the Low RS (L) wine base; another one was a 2012 late harvest Riesling, alcohol content 9.6%, residual sugar 64.1 g/L, defined as the High RS (H) wine base. Wine (100 mL) was de-aromatized using a rotary evaporator (Buchi heating bath B-490, Newcastle, DE, USA) at 35 °C water bath and 135 rpm rotation for 2 h under 63 cmHg vacuum. The dearomatized wine was adjusted with ethanol solution to its original alcohol content and dH_2_O was added to bring the volume up to the original 100 mL, ensuring the same nonvolatile matrix. The dearomatized wine contained only trace amounts of linalool oxide and α-terpineol isomers. These concentrations were subtracted to from the calibration curves to ensure accurate measurements. 

Six point calibration curves for all compounds were plotted. Compounds were added to de-aromatized wine base at specific concentrations (Table 6) in 20 mL amber glass, screw cap vials, 22.5 × 75.5 mm, followed by the composite isotopically-labelled internal standard solution (40 µL), to make a final volume of 9.0 mL. 

Over the lifetime of the SPME fiber, the partition coefficients of volatile compounds with the SPME fiber may have varied thus resulting in poor fiber repeatability. Calibration curves were divided into three parts at the beginning of a new fiber, middle use of fiber and at the last life of fiber. In total six kinds of calibration curves were plotted in terms of wine matrix and fiber over time. Each calibration curve was generated in triplicate (Table 6).

#### 3.5.2. Limit of Detection (LOD), Limit of Quantitation (LOQ), Wine Reproducibility and Internal Standards Stability

LOD and LOQ (Table 2) were calculated as in [56] and [57]. Two wine samples (2011 dry Riesling, ALC of 10.8%, RS of 0.58 g/L; 2013 Pinot Gris, ALC of 11.8%, RS of 16.43 g/L) were measured every 2 days over the course of the analysis period to determine stability and reproducibility. The wine samples were all kept frozen (at −18 °C) prior to analysis. The Standard 4 point of the calibration curve was made fresh and measured every 2 days over the course of the analysis period to measure internal standard stability (Table 2).

**Table 6:** Six point concentrations of each compound for calibration.

Compound	Standard 1(µg/L)	Standard 2 (µg/L)	Standard 3 (µg/L)	Standard 4 (µg/L)	Standard 5 (µg/L)	Standard 6 (µg/L)
2	0.00	0.65	1.29	2.58	5.16	7.74
4	0.00	0.77	1.52	3.04	6.08	9.12
5	0.00	0.04	0.08	0.17	0.34	0.50
6	0.00	0.17	0.33	0.66	1.32	1.98
7	0.00	3.06	6.09	12.14	24.32	36.46
8	0.00	3.17	6.31	12.58	25.20	37.78
9	0.00	0.06	0.12	0.23	0.47	0.71
10	0.00	0.02	0.04	0.09	0.18	0.27
11	0.00	2.33	4.64	9.25	18.53	27.77
12	0.00	2.49	4.95	9.86	19.75	29.62
14	0.00	2.45	4.88	9.72	19.48	29.20
16	0.00	2.36	4.69	9.34	18.71	28.05
18	0.00	2.88	5.72	11.41	22.86	34.28
20	0.00	4.80	9.55	19.05	38.15	57.19
21	0.00	1.02	2.03	4.05	8.12	12.17

#### 3.5.3. Accuracy

The accuracy of the analytical method was evaluated by calculating the recoveries (Table 2) of the standard addition. Different styles of wines (dry, medium dry, medium sweet and sweet) were selected, each of them spiked to standard four concentrations. The spiked recoveries were calculated by the difference of concentration of compounds between spiked wine and original wine divided by the real concentration of compounds in standard 4. The two kinds of varieties (Riesling and Pinot Gris) were investigated, respectively, in duplicate. 

#### 3.5.4. Temperature Stability

Seven chiral monoterpenes, (+)-α-terpineol, (−)-linalool, (*R*)-(+)-β-citronellol, (*R*)-(+)-limonene, (*S*)-(−)-limonene, (+)-(2*R*,4*S*)-*cis*-rose oxide, and (−)-(2*S*,4*R*)-*cis*-rose oxide were chosen to detect temperature stability. Each compound was added to milli-Q water at concentrations close to standard 6 and extracted with SPME at 60 °C for 50 min; the purity (peak area ratio of these compounds to corresponding isomers) was investigated at injector temperature of 200 °C, 230 °C and 250 °C, respectively. The extraction temperature was performed at 40 °C and 60 °C as injector temperature at 250 °C.

### 3.6. Chiral Mono-Terpene Contents in 12 White Wines

Twelve white wines from Riesling and Pinot Gris grape varieties were donated from top companies all over the world, including New Zealand, Australia, Italy and USA. Detailed information about the 12 wines is listed in Table 7. Wine type was categorized according to European regulation [58]: wine with residual sugar content not exceeding 4 g/L can be considered as “dry”; between 4 g/L and 12 g/L as “medium dry”; not exceeding 45 g/L as “medium sweet”, above 45 g/L as “sweet” wine. All of the wines were sampled and stored at −18 °C before analysis. 

**Table 7:** Riesling and Pinot Gris white wine samples from different regions.

Wine Code	Vintage	Region	Sub-Region	Alcohol Content (*v*/*v*)	Residual Sugar Content (g/L)	Wine Type [40]
P1	2011	Italy	Friuli Grave	12.50%	0.86	Dry
P2	2013	Oregon	Willamette Valley	13.17%	2.68	Dry
P3	2012	Oregon	Willamette Valley	13.89%	3.95	Dry
P4	2013	Oregon	Willamette Valley	12.62%	4.23	Medium dry
P5	2013	Australia	Limestone Coast	14.06%	5.49	Medium dry
P6	2013	New Zealand	Auckland	12.91%	7.41	Medium dry
R1	2013	Australia	Eden valley	11.63%	3.72	Dry
R2	2012	Oregon	Willamette Valley	13.17%	2.68	Dry
R3	2012	Washington	Columbia Valley	12.92%	5.69	Medium dry
R4	2012	Washington	Yakima valley	12.52%	15.00	Medium sweet
R5	2012	New York	Finger lakes	11.43%	15.27	Medium sweet
R6	2013	Washington	Columbia Valley	7.07%	95.83	Sweet

### 3.7. Data Analysis

T-test, ANOVA and Tukey multiple comparison were calculated with XLSTAT-Pro 2014 (Addinsoft). Principle Component Analysis (PCA) using Pearson correlation matrix was also calculated with XLSTAT-Pro 2014 (Addinsoft, New York, NY, USA). 

## 4. Conclusions 

It is possible to measure different monoterpene enantiomers in white wine using an HS-SPME-MDGC-MS method. This method was able to quantify concentrations of the different enantiomers down to 1 ng/L. It is not possible to use a single calibration curve for all wines as a significant matrix effect, related to sugar content, was noted. Additionally, the fiber age also impacted the measurement of some of the chiral terpenes. In particular the rose oxide isomers, and limonene isomers were most impacted by fiber life and the matrix. However this method resulted in good separation and sensitive, accurate and reproducible measurement of chiral monoterpenes when different wine matrixes were used. A difference in chiral monoterpene content was noted between Pinot Gris and Riesling wines, with Riesling wines containing a larger number of the chiral terpenes than Pinot Gris wines. Terpenes are known to impact the floral and citrus aromatics of wine, but the identification and quantitation of the individual isomers may explain differences in these aromatics between wines. Further investigations will determine if the different terpene isomers impact wine aroma.
